# Minimal Long-Term Neurobehavioral Impairments after Endovascular Perforation Subarachnoid Hemorrhage in Mice

**DOI:** 10.1038/s41598-017-07701-y

**Published:** 2017-08-08

**Authors:** Claudia Fanizzi, Andrew D. Sauerbeck, Mihika Gangolli, Gregory J. Zipfel, David L. Brody, Terrance T. Kummer

**Affiliations:** 1 Department of Neurology, Washington University School of Medicine in St. Louis, Missouri, USA; 2 Department of Neurosurgery, Washington University School of Medicine in St. Louis, Missouri, USA; 3 Department of Neurosurgery, Fondazione IRCCS Ca’ Granda Ospedale Maggiore Policlinico, Milan, Italy

## Abstract

Cognitive deficits are among the most severe and pervasive consequences of aneurysmal subarachnoid hemorrhage (SAH). A critical step in developing therapies targeting such outcomes is the characterization of experimentally-tractable pre-clinical models that exhibit multi-domain neurobehavioral deficits similar to those afflicting humans. We therefore searched for neurobehavioral abnormalities following endovascular perforation induction of SAH in mice, a heavily-utilized model. We instituted a functional screen to manage variability in injury severity, then assessed acute functional deficits, as well as activity, anxiety-related behavior, learning and memory, socialization, and depressive-like behavior at sub-acute and chronic time points (up to 1 month post-injury). Animals in which SAH was induced exhibited reduced acute functional capacity and reduced general activity to 1 month post-injury. Tests of anxiety-related behavior including central area time in the elevated plus maze and thigmotaxis in the open field test revealed increased anxiety-like behavior at subacute and chronic time-points, respectively. Effect sizes for subacute and chronic neurobehavioral endpoints in other domains, however, were small. In combination with persistent variability, this led to non-significant effects of injury on all remaining neurobehavioral outcomes. These results suggest that, with the exception of anxiety-related behavior, alternate mouse models are required to effectively analyze cognitive outcomes after SAH.

## Introduction

Subarachnoid hemorrhage (SAH) from the rupture of an intracranial aneurysm is the most devastating variant of vascular brain injury. SAH carries a 1-month mortality rate of approximately 40%^1, 2^, and survivors are often burdened with substantial neurological injuries. Unlike the characteristically focal motor and sensory deficits that result from most vascular brain injuries, the primary difficulty reported by SAH survivors is neurobehavioral impairment^3^. Abnormalities in learning and memory, executive function, attention, anxiety, mood, and socialization are found in 75% of SAH survivors^4–7^, and reported by patients to interfere with daily activities at even higher rates (95% in one study)^8^.

The mechanisms underlying post-SAH cognitive impairments are poorly-understood. Partly as a result, there are no therapies proven to improve cognitive outcomes after SAH. Development of experimentally-tractable SAH animal models that exhibit multi-domain neurobehavioral deficits could help to both reveal causative mechanistic pathways, and evaluate their interruption with targeted therapeutics.

Animal models of SAH differ in species and in method of induction^9^, each with their own strengths and weaknesses. Mice are a particularly attractive model organism due to their low carrying costs, short generational time, and the deep library of genetic models that have been developed and characterized to query specific mechanistic pathways. This is directly relevant to SAH. For example, apolipoprotein E genotypes are independently associated with poor post-SAH cognitive outcomes^10, 11^. Although long-term learning and memory deficits have been demonstrated in all three primary models of SAH in rats^12–18^, most consistently in Morris water maze (MWM) testing, similar deficits have not been reported using any of the dominant methods of inducing SAH in mice. Alternate long-term emotional, social, and cognitive effects, despite their prominence in human SAH-induced cognitive impairment, have similarly not been reported.

We recently found that endovascular perforation, a heavily-utilized SAH model with high face-validity, fails to cause MWM deficits in mice^19^. A post-hoc analysis of this data focusing on more severely-injured animals (neuroscore at 1 day post-injury <23), however, did reveal a statistically-significant effect of injury (data not shown). This is consistent with known variability in injury severity that occurs after endovascular perforation^9, 20^. In an effort to develop this model for mechanistic analysis and amelioration of multi-domain post-SAH cognitive impairment, we sought to determine whether multi-domain neurobehavioral deficits can be detected with rigorous blinding across multiple independent cohorts in a group consisting of all mice subjected to endovascular perforation SAH, or in a moderate- to severely-injured subgroup.

## Results

### Experimental protocol and prescreening

**Figure 1:**
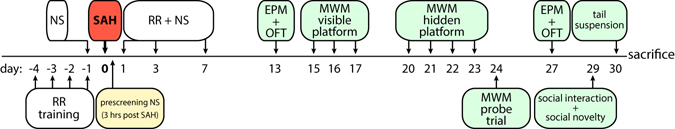
Experimental timeline.

**Figure 2:**
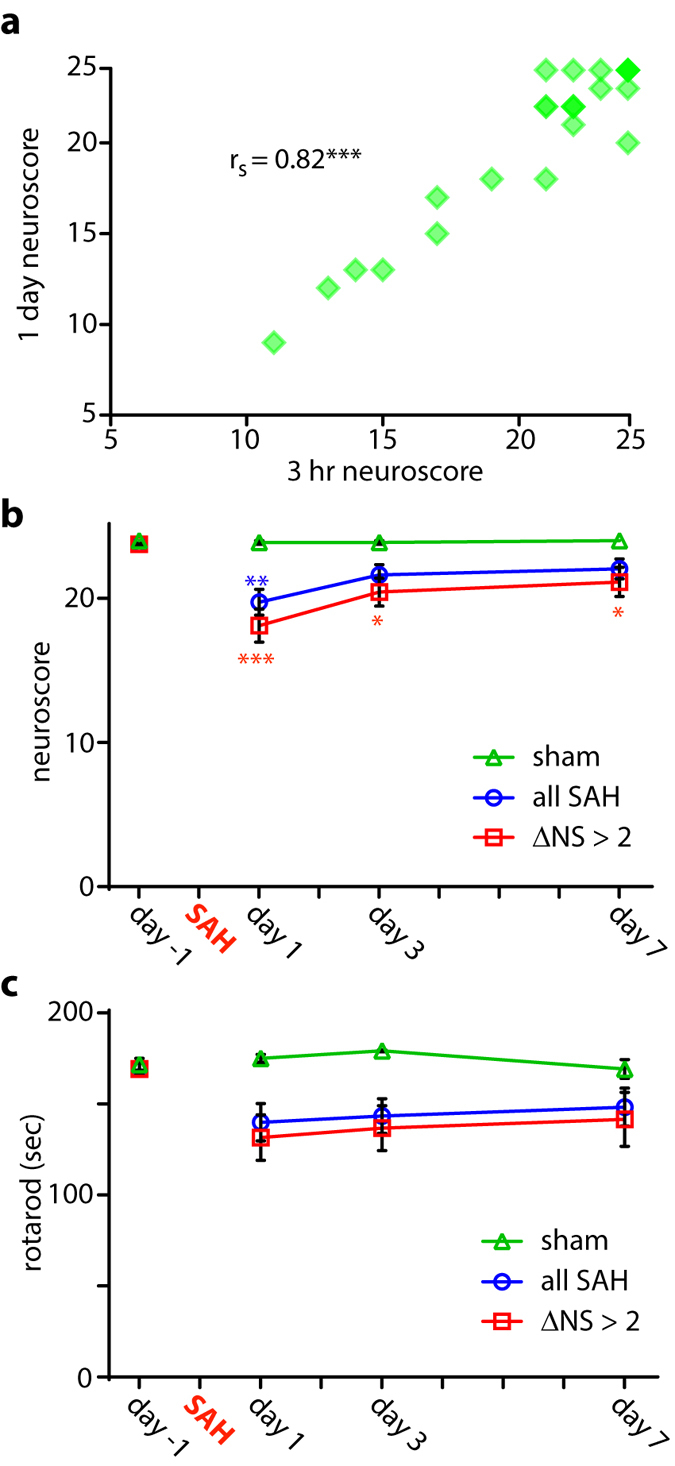
Acute and sub-acute sensorimotor impairment. Neuroscore (NS) collected 3 hours after injury compared with that measured at 1 day post-injury (**a**). NS (**b**) and rotarod (**c**) evaluation one day before and at days 1, 3, and 7 post-injury. Only animals meeting inclusion criteria (NS > 22 and RR ≥ 140) are depicted. Spearman correlation (**a**). Mann-Whitney U test with Holm-Bonferroni correction for multiple comparisons (**b**,**c**). RR before correction for multiple comparisons; day 1: *P* = 0.30 sham vs. all SAH, *P* = 0.14 sham vs. ΔNS > 2; day 3: *P = *0.06 sham vs. all SAH, *P* = 0.05 sham vs. ΔNS > 2; day 7: *P* = 0.63 sham vs. all SAH, *P* = 0.54 sham vs. ΔNS > 2.

The experimental time line is depicted in Fig. 1. Three independent cohorts of 15 C57Bl/6 mice, all containing sham and injury groups, were used for these experiments. To limit variability in injury severity and functional performance, mice were prescreened in two stages. We first eliminated poor performers using the rotarod (RR) and neuroscore (NS) tests prior to injury (average RR latency <140 seconds and/or NS ≤ 22 not studied further). Of the original 45 animals, this left 37 (18% eliminated) for testing. At 3 hours following injury, animals again underwent NS testing for group assignment purposes. Mice subjected to endovascular perforation but with a change in NS of ≤2 from that animal’s baseline were considered to have very mild injuries; those with a change in NS > 2 were considered to have moderate-severe injury. The 3-hour time point was chosen to permit future assessments of therapeutics in a clinically-relevant window post-injury without the potential for treatment to confound the group assignment process. We found that NS at 3 hours correlated well with that measured at 24 hours (Spearman’s r_s_ 0.82; Fig. 2a), the time point used for prescreening in our prior post-hoc subgroup analysis of MWM performance. For all following analyses we considered both the injury group containing all animals subjected to SAH and the subgroup with moderate-severe injuries. Of the 37 animals injured, 28 survived to completion of neurobehavioral testing (mortality rate 28% after SAH).

### Acute and sub-acute sensorimotor impairment

As expected^21^, animals subjected to SAH exhibited acute decrements in NS and RR performance that improved by the end of the first week post-injury (Fig. 2b and c). The group containing all animals subjected to SAH was statistically indistinguishable from shams on NS and RR performance by day 3 post-injury, while those in the moderate-severe group (change in NS at 3 hours > 2) retained a small but statistically significant deficit on NS testing only compared to shams. There was a trend towards reduced RR performance in both injury groups at early time points that was less evident by day 7 post-SAH.

### General activity

**Figure 3:**
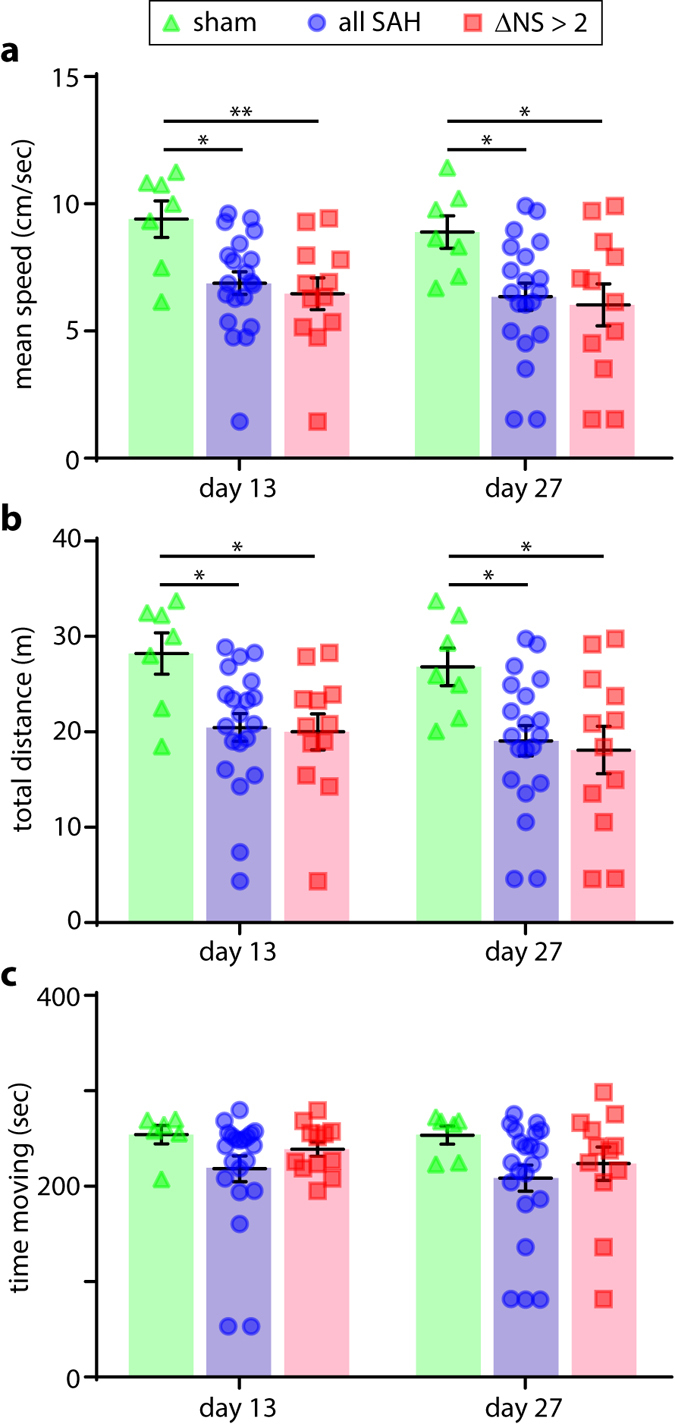
General activity assessments. Assessment of mean speed (**a**), total distance (**b**), and time spent moving (**c**) during passive exploration in the open field test at 13 and 27 days post-injury. t-test with Holm-Bonferroni correction for multiple comparisons (**a**). Mann-Whitney U test with Holm-Bonferroni correction for multiple comparisons (**b**,**c**). Time moving before correction for multiple comparisons; day 13 *P = *0.17 sham vs. all SAH, *P* = 0.48 sham vs. ΔNS > 2; day 24 *P* = 0.05 sham vs. all SAH, *P* = 0.34 sham vs. ΔNS > 2.

We examined overall activity in the open field test (OFT) as a gauge of general neurological function at time-points beyond 7 days post-injury. The OFT was utilized in lieu of more stressful sensorimotor testing (*i.e*., NS and RR) to avoid confounding concomitant neurobehavioral endpoints. Interestingly, although testing of stimulated function (NS/RR) demonstrated minimal differences by the end of the first week, spontaneous activity remained significantly lower in animals subjected to SAH vs. sham controls out to 27 days post-injury, as measured by mean speed (Fig. 3a) and total distance (Fig. 3b). There was also a trend towards less time spent moving after SAH vs. controls (Fig. 3c) at these later time points. Effect sizes were similar whether the sham-injured animals were compared to all SAH animals or only the moderate-severe subgroup.

### Anxiety- and mood-related behaviors

**Figure 4:**
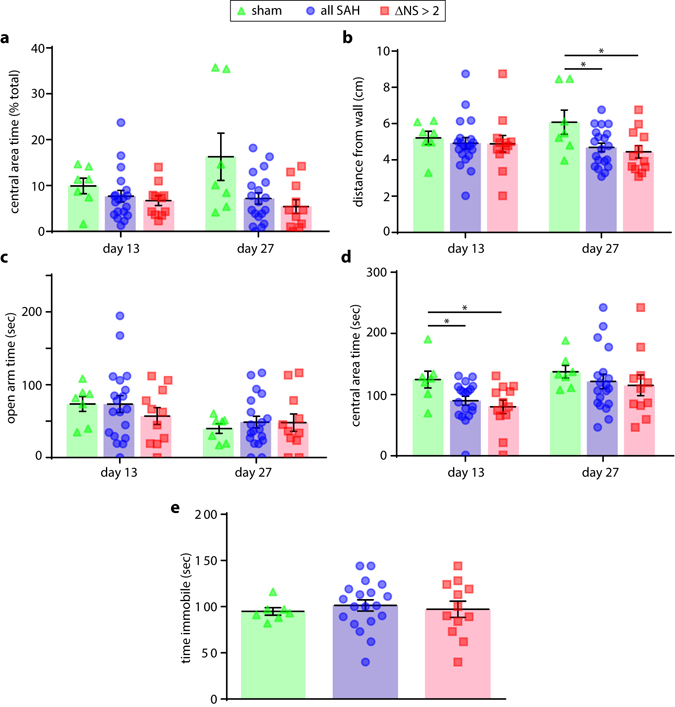
Anxiety and mood-related behaviors. Thigmotaxic behavior was quantified in the open field test by evaluating the percent time spent in a central region (**a**) and by calculating the average distance from the nearest wall at 13 and 27 days post-injury (**b**). Time spent in the open area (**b**) and central area (**c**) of the elevated plus maze was quantified. Time immobile during the tail suspension test (**d**). Mann-Whitney U test with Holm-Bonferroni correction for multiple comparisons (**a**–**c**). t-test with Holm-Bonferroni correction for multiple comparisons (**d**,**e**).

Two neurobehavioral tests were employed to examine anxiety-related behavior after SAH at subacute and chronic time points. Thigmotaxis, the tendency of mice to explore primarily near walls^22^, is a well-studied model of anxiety-related behavior with high pharmacological predictive validity^23^. Thigmotaxic behavior was initially quantified in the OFT by measuring the percent time spent in a central region (central 30% of the testing box). A non-significant trend was observed towards less time spent exploring the central area in injured animals that was more prominent on day 27 (Fig. 4a). To eliminate the arbitrarily-sized central region as a factor in the analysis, we calculated the mean distance from the nearest wall throughout the exploratory period and again compared groups. Interestingly, with this analysis on day 27 post-injury control animals explored significantly further from the wall than either injured group, while thigmotaxic behavior differed minimally between groups 13 days post-injury (Fig. 4b).

To corroborate these results, the same animals were tested in the elevated plus maze (EPM), which evaluates an animal’s relative tendency to leave the safety of walled enclosures (the closed arms) for more open areas (the central area and open arms)^24^. No significant differences in time spent on the open arms was observed between groups at either time point (Fig. 4c). Injured animals did, however, spend significantly less time in the central area at 13 days post-injury (Fig. 4d). This effect did not reach statistical significance on day 27.

Thus both tests (OFT, EPM) contained indications of increased anxiety-related behavior, but these effects were limited to certain endpoints and did not manifest at all post-injury intervals. Differing repeated measures effects with these test protocols as well as inter-test interactions may be partly responsible (see discussion). The tail suspension test, in which an animal is suspended by its tail and the time immobile is quantified^25, 26^, was used to assess depressive-like behavior at a single time-point 30 days post-injury. Total time immobile is a pharmacologically-validated surrogate for depressive symptomatology^27^: animals with greater depressive-like symptoms will spend relatively less time struggling. As tail suspension is a stressful test, to avoid interaction with other behavioral measures it was assessed only at a single time-point after all other tests were complete. No statistically-significant differences between groups were noted in time immobile (Fig. 4d), indicating a relative lack of depressive-like symptoms following SAH in this model at both injury severities.

### Learning and Memory

**Figure 5:**
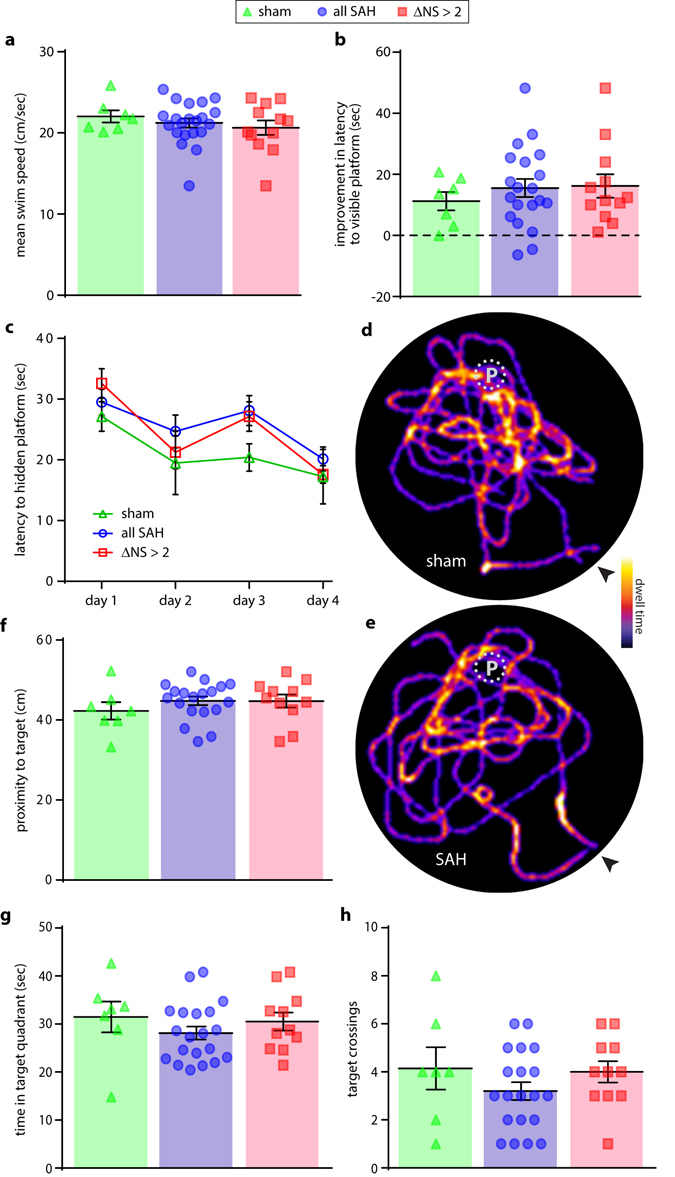
Learning and memory. Measurement of mean swim speed in the Morris water maze visible platform test (**a**). Improvement in time to reach a visible platform was quantified during cued training over 3 days (**b**). Performance over 4 days of hidden platform training was monitored (**c**), followed by a single probe test in which the platform was removed and the swim pathways (**d**,**e**) two representative paths are show for each group; platform indicated with P; point of insertion indicated with arrowhead), average distance from the target (**f**), time in the quadrant of the pool containing the target platform (**g**), and number of platform crosses (**h**) were monitored and quantified. t-test with Holm-Bonferroni correction for multiple comparisons (**a**,**b**,**f**–**h**). 2-way repeated-measures ANOVA (**c**).

The Morris water maze test was used to evaluate learning and memory post-SAH. Visible platform testing (cued testing) did not reveal differences in swimming ability, motivation, or ability to acquire the task (Fig. 5a,b). All groups of animals exhibited improvement in latency to target over four days of hidden platform testing (Fig. 5c). There were no statistically-significant differences between groups at any time point, irrespective of injury severity. The day following the final trial of hidden platform training (post-injury day 24) mice were subjected to a 60 second probe trial to test memory with the platform removed. A qualitative inspection of swim pathways during the probe trial revealed only subtle differences in swim patterns, with no clear distinctions in terms of search strategy (Fig. 5d,e). Quantitation demonstrated similar performance in this task, with no statistically-significant difference in proximity to target between groups (Fig. 5f). Time in target quadrant and number of target crossings were also not found to differ significantly between groups (Fig. 5g,h).

### Sociability

**Figure 6:**
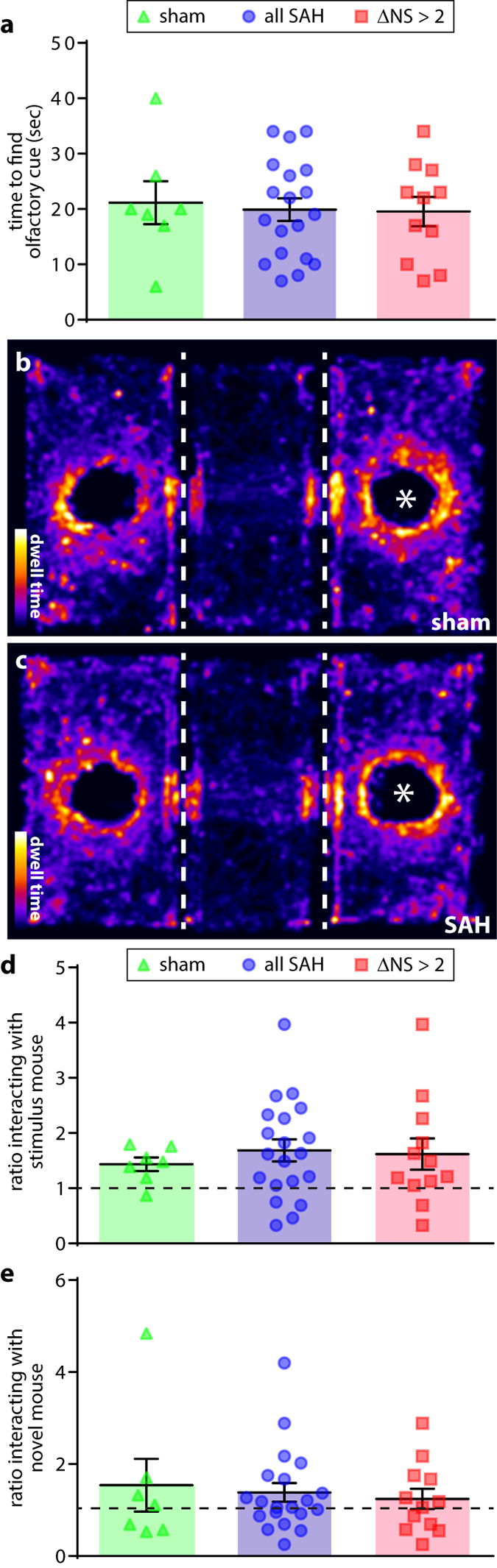
Social interaction and social novelty/social memory. Measurement of time to find a buried olfactory cue (**a**). Heat maps of dwell time in the 3-chamber social interaction test (**b**,**c**). Dotted lines indicate chamber divisions. Asterisk indicates location of stimulus mouse (dummy mouse placed into opposite chamber). Quantification of ratio of time spent interacting with stimulus mouse vs. dummy mouse (**d**), dotted line indicates no preference). Ratio of time spent interacting with novel stimulus mouse vs. original stimulus mouse (**e**), dotted line indicates no preference). t-test with Holm-Bonferroni correction for multiple comparisons (**a**,**d**). Mann-Whitney U test with Holm-Bonferroni correction for multiple comparisons (**e**).

Social interaction (SI) and social novelty (SN)-seeking behaviors/social memory were assessed using Crawley’s sociability and preference for social novelty protocol^28^. A control assessment of olfaction (buried cereal test) revealed no statistically-significant differences between groups (Fig. 6a). Following habituation in a 3-chamber SI/SN apparatus, animals were allowed to freely interact with either an unfamiliar stimulus mouse or a dummy mouse. After this trial, the dummy mouse was replaced with a novel stimulus mouse and the test was repeated. Heat maps of dwell time demonstrated a preference for the stimulus mouse in all groups (Fig. 6b,c). Quantification similarly revealed that all groups exhibited comparable social behavior (Fig. 6d). Subsequent analysis of behavior in the social novelty test furthermore failed to reveal group-wise differences in social novelty seeking/social memory (Fig. 6e).

### Cerebral volumetrics

**Figure 7:**
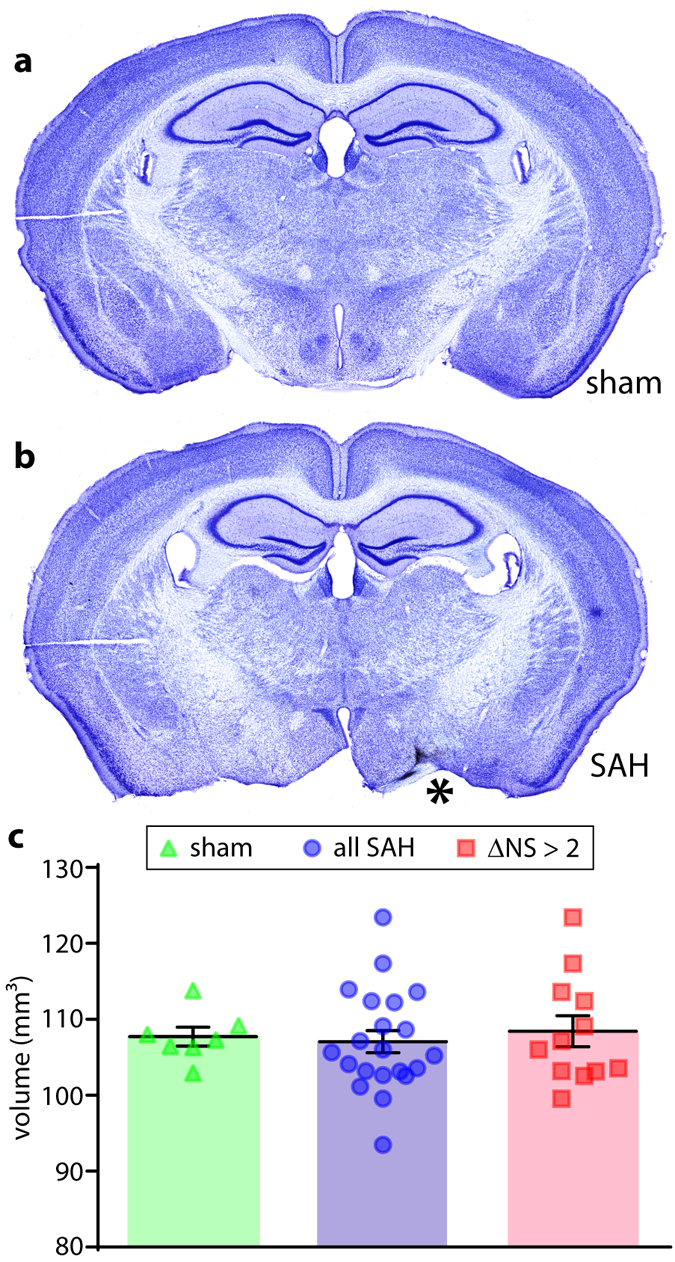
Cerebral volumetrics. Cresyl violet-stained sections from a representative sham-injured animal (**a**) and an animal subjected to endovascular perforation SAH (**b**; *site of arterial rupture). Brain parenchymal volume was quantified over a 2.7 mm antero-posterior portion of the brain centered near the depicted sections for sham-injured animals and both SAH groups (**c**). Mann-Whitney U test with Holm-Bonferroni correction for multiple comparisons (**c**).

To evaluate the development of atrophy as a potential correlate of neurobehavioral deficits in the endovascular perforation SAH model, we calculated cerebral volumetrics for an anatomically-restricted antero-posterior portion of the brain containing the vascular rupture site. Though focal lesions near the site of arterial rupture were frequently observed (Fig. 7a,b), this analysis did not reveal evidence of atrophy at 1 month post-injury at either SAH-severity compared to sham-treated animals (Fig. 7c).

### Prescreening neuroscore and cerebral volume fail to predict neurobehavioral endpoints

Lastly we determined whether the 3-hour NS or cerebral volume at 30 days were able to predict behavioral outcomes across the range of neurobehavioral tests employed. No correlation coefficient exceeded ±0.4, and all were statistically non-significant before correction for multiple comparisons (data not shown).

## Discussion

Cognitive, emotional, and social impairments are a debilitating and highly-prevalent feature of aneurysmal subarachnoid hemorrhage^3–8^. Consequently, despite representing 3–5% of all stroke, SAH accounts for 27.3% of stroke-related years of productive life lost—a burden on par with that of ischemic stroke (38.5%) and all other variants of hemorrhagic stroke (34.2%)^2^. Development of effective therapies for ameliorating these impairments would be facilitated by the characterization of a mouse model in which they are faithfully recapitulated.

Two further observations underline the importance of this approach: (1) The developing field of early brain injury is uncovering a number of novel pathophysiological processes such as oxidative stress, inflammation, microvascular thrombosis, apoptosis and axonal injury that may contribute to cognitive impairment after SAH^21, 29–31^. (2) Recent therapeutic trials successfully targeting traditional, short-term outcomes such as cerebral vasospasm^32^ have failed to yield lasting benefits for patients. Animal models of post-SAH neurobehavioral impairments will be essential to evaluate novel injury mechanisms in terms of the long-term cognitive outcomes with the greatest salience for patients^33^. The rich toolset of genetically modified mice makes this species particularly attractive for such mechanistic analysis.

We measured a battery of neurobehavioral endpoints out to 1 month post-injury in a heavily-utilized mouse model of subarachnoid hemorrhage, the endovascular perforation model. Although this model has desirable face validity compared to cisternal blood injection, it suffers from greater experimental variability in injury severity. We attempted to moderate this through the use of pre-injury and immediate post-injury screening/stratification.

While measures of sensorimotor activity very nearly normalized by 7 days post-injury, deficits in general activity at both severity thresholds compared to sham-injured animals were observed 1 month after injury. We also noted increased thigmotaxic behavior in injured animals 1 month after SAH when analyzed using a distance-to-wall measurement. These are, to our knowledge, the longest-lasting behavioral phenotypes documented for the endovascular perforation mouse model. Altered performance in the OFT has previously been found at acute^34, 35^ and sub-acute time points: Li and colleagues recently reported altered OFT thigmotaxic behavior at 14 days post-injury in the endovascular perforation model in mice^36^. Interestingly, they observed a significant decrease in thigmotaxic behavior following SAH compared to sham at day 14, whereas we observed no differences in thigmotaxic behavior at this time point, and an increase in thigmotaxis thereafter. Several explanations, including differences in injury model (single vs. double perforation) and OFT methodological parameters, may explain this discrepancy. Differential, model-dependent effects in another anxiety-related behavioral phenotype after SAH—open arm time in the EPM—have similarly been found in rats^12, 18^.

Evidence of anxiety-related behaviors were also observed in the EPM, however these assessments did not reach statistical significance for endpoints including open arm time, and must therefore be interpreted with caution. Use of an elevated zero maze, in which the central area is absent, would be a useful method to eliminate ambiguity between the open arms and the central area in the EPM. Curiously, anxiety-related behaviors were most prominent in the EPM at 2 weeks, while they became significant in the OFT only at 1 month. The EPM is known to have a prominent repeated measures effect, with fewer open arm entries and less time in the central area on subsequent assessments^24^. This may explain the lack of anxiety related behavioral changes upon repeat assessment at 1 month in the EPM in this study. Inter-test interactions may also contribute. We carefully structured behavioral assessments to limit test-test interactions as much as possible (stressful tests conducted at the end or separated by many days from other tests). Future studies would ideally stratify a smaller number of behavioral endpoints into individual cohorts for each assessment.

Importantly, we failed to demonstrate a behavioral phenotype for the remainder of the assessed neurobehavioral domains. The potential explanations for this include several factors. First, though we took many steps to limit variability both in our injury model and in our behavioral assessments, as the scatter plots in Figs 3–7 make clear, considerable variability in outcomes persists. Indeed, injury severity had almost no effect on long-term outcomes or on the variability of those outcomes. One possibility is that, absent ICU-level care post-SAH, the animals that would manifest long-term neurocognitive deficits do not survive the injury. It is also potentially relevant that sham-injured animals in this model are not entirely free from cerebral injury. We have previously observed changes in diffusion and T2 MRI signal intensity in the week following sham endovascular perforation in mice^21^ (and data not shown), presumably due to alterations in cerebral blood flow resulting from manipulation of neck vasculature. The range of detectable injury may be restricted due to mild impairment in the control group. We did not include naïve controls in these experiments to directly test this hypothesis. Lastly, the endovascular perforation model of SAH in mice may lack the extent and/or distribution of neuronal injury, death, or dysfunction necessary to manifest neurobehavioral deficits beyond the acute period when general cerebral dysfunction is marked. Cell death has been documented in the ipsilateral parietal cortex^37^ and ipsilateral basal cortex^38^ in this model, but we and others previously failed to detect cell or volume loss in the hippocampus^19, 39^. In contrast, ipsilateral hippocampal atrophy and apoptosis are both observed following SAH in humans^40, 41^. It is furthermore important to note that, while our battery of neurobehavioral assessments focused on well-validated tests that encompass several domains of cognitive impairment post-SAH in humans, it remains possible that more sensitive or specific behavioral assays will detect additional long-term impairment following endovascular perforation SAH.

Given the difficulties involved in studying long-term neurocognitive disability in the endovascular perforation model in mice, other models should be considered for the analysis of these endpoints. Cisternal injection, the second major murine model of SAH, has similarly not been shown to exhibit long-term neurobehavioral deficits^18, 42^. Altay and colleagues introduced a model involving exposure and transection of a consistently-identified intracisternal vein via the atlanto-occipital membrane^43^. This group recently reported MWM deficits using this model at 34 days post-injury^44^. Interestingly, neutrophil depletion largely rescued SAH-induced MWM deficits. Thus this method represents a promising avenue for the exploration of learning and memory after SAH. Sabri and colleagues introduced a prechiasmatic blood injection murine model of SAH^45^, but behavioral characterization of this model is currently limited to the acute period (7 days post-injury) and to primarily sensorimotor assessments^46^.

Strengths of our approach include a multiple, independent cohort design, rigorous blinding to experimental groups through the analysis phase, careful control of visual, acoustic, olfactory, and diurnal variables during behavior testing, automated analysis for all behavioral outcomes except tail suspension, and the use of multiple measures to limit variability inherent in the model itself. Limitations of our approach include the performance of several functional and behavioral tests in the same animals. As noted above, steps were taken to limit the effect of inter- and intra-test interactions, but these can never be fully eliminated. Although we carried our testing to 1 month post-injury, a common endpoint for long-term neurobehavioral studies, standards are shifting towards 3, 6, or even 12 months in some situations. Knowledge of the injury process and of the temporal evolution of the selected endpoints in humans should guide the planning of future preclinical SAH studies. Lastly there are many additional potential neurobehavioral tests that could be explored. We selected this battery to cover those frequently employed in the field of preclinical SAH research as well as in related fields, to represent several different domains of neurocognitive functioning impaired after human SAH, and to maintain harmony between tests.

In conclusion, we find that the mouse endovascular perforation model of SAH results in minimal long-term neurobehavioral deficits, with the exception of anxiety-related behaviors, which are only apparent in some endpoints. Although murine endovascular perforation may prove to be a useful method to analyze anxiety post-SAH, future studies seeking to mechanistically explore the broader landscape of cognitive impairment in the hope of developing targeted therapeutics should consider alternate models.

## Methods

### Animals

All experimental protocols were approved by the Animal Studies Committee at Washington University School of Medicine in St. Louis and carried out in accordance with relevant guidelines and regulations. Male C57BL/6 wild-type mice were purchased at 11 weeks of age from Jackson Labs (Bar Harbor, ME, USA), housed in an AAALAC-accredited, pathogen-free facility with access to standard chow and water *ad libitum*, and monitored at least daily by a trained veterinarian.

### Endovascular perforation

All injuries were performed by a single technician who was not involved in data collection or analysis. Allocation to experimental group was performed by this technician just prior to injury. All study investigators were kept blind to experimental group until the end of data collection and analysis. Endovascular perforation SAH was performed as described^47^ with slight modification. Briefly, 12 week-old mice were anesthetized with isoflurane (2% induction, 1.5% maintenance) and a 5–0 nylon suture was introduced into the left external carotid artery and advanced through the internal carotid artery until reaching the left internal carotid artery bifurcation. The suture was advanced 3 mm to induce arterial rupture, withdrawn 3 mm and re-advanced 3 mm to induce a severe hemorrhage. The suture was then removed allowing reperfusion and SAH, and the external carotid artery was ligated. Randomly-selected littermates underwent sham injury consisting of the same procedure except that the suture was not advanced past the internal carotid artery bifurcation. Twice daily on days 0–3 mice were administered 50 mg/kg ampicillin (Sigma-Aldrich, St. Louis, MO, USA) in 0.5 mL of a 10% dextrose solution in sterile saline^19^. On post-operative days 4–7 mice were administered 0.5 mL of 10% dextrose in saline twice daily. After 7 days the animals were anesthetized with 2% isoflurane and the stiches were removed.

### Acute functional testing and prescreening

Rotarod (RR): To minimize an effect of learning on RR performance post-injury, animals were pre-trained on the RR (Rotamex Columbus Instruments, Columbus, OH, USA) for 4 days prior to injury using a protocol identical to post-injury testing. Speed was set to accelerate 0.1 rpm/sec from 2 to max 20 rpm (3 cm rod diameter) over a trial length of 180 seconds. Each trial was repeated three times per day and the latency to fall was averaged. Mice were assessed by RR on post-injury days 1, 3, and 7.

Neuroscore (NS): NS was determined as described^47, 48^ one day prior to injury, three hours after injury, and on post-injury days 1, 3, and 7. Neurological function was graded based on a motor score (0 to 12) that assessed spontaneous activity, symmetry of limb movements, climbing, and balance and coordination, and on a sensory score (4 to 12) that assessed body proprioception and vibrissae, visual, and tactile responses.

Prescreening: Mice were prescreened one day prior to injury to eliminate poor performers (average RR latency <140 seconds and/or NS ≤ 22), and again 3 hours after injury to identify very mild injuries (NS change of ≤2 from baseline) for subgroup analysis. All injured animals underwent the full functional and behavioral battery. Injured mice were analyzed in two groups, the full group of SAH animals including those with a change in NS > 2 from baseline, and the subgroup of mice with a change in NS > 2 from baseline (the moderate-severe SAH group).

### Neurobehavioral testing

All neurobehavioral testing was carried out in a dedicated behavior room located within mouse housing facilities during workday hours. This room was equipped with light, sound and humidity controls and isolated from external noise. Mice were allowed to acclimate in this room for at least 30 min prior to testing. Illuminance was measured and set independently at the start of each test (40 lux for Morris water maze testing; 20 lux for all other tests) using a lux meter (Sper Scientific 840006, Scottsdale, AZ, USA). A white noise machine (Marpac Dohm-DS, Wilmington, NC, USA) was set to deliver 60 dB at the test apparatus, measured with a sound level meter (Lafayette Instrument SL-A, Lafayette, IN, USA). To eliminate scents, all testing surfaces were cleaned with 70% ethyl alcohol prior to testing and between animals. An overhead camera recorded all mouse paths, which were subsequently analyzed using SMART analysis software (San Diego Instruments, San Diego, CA, USA). Heat maps of dwell time were prepared for the Morris water maze and social interaction/social novelty tests by exporting coordinate data from SMART, processing these coordinates into individual heat maps using a custom MATLAB script (Mathworks, Natick, MA, USA), overlaying individual heat maps by group using Photoshop (Adobe, San Jose, CA), then transforming to a color look-up table in ImageJ (NIH, Bethesda, MD, USA).

Open field test (OFT): On post-injury days 13 and 27 animals were released into the same corner of a 44.5 × 44.5 cm white plastic box and allowed to freely explore for 5 min. Distance traveled, average speed, and time spent moving were measured. To quantify thigmotaxic behavior, the box was subdivided into concentric zones separated by 1.5 cm and starting 1.5 cm from the wall of the box (15 squares in total). We then calculated the time spent in the central 30% of the box and the average distance from the nearest wall throughout the trail. To calculate average distance from the wall, the fraction of time spent in each 1.5 cm-wide zone was multiplied by its distance from the wall and the resulting products summed.

Elevated Plus Maze (EPM): On post-injury days 13 and 27 animals were released into the closed arm of a custom elevated plus maze (30 cm arm length, 5 cm arm width, elevated 50 cm above board) and allowed to explore freely for 5 min. Open arms were fit with a 2 mm guard rail to prevent falls. Time spent in open and closed arms and in the central area (defined as a box 14 × 14 cm centered on the maze center) was measured using the animal’s center of mass as a reference in SMART.

Morris Water Maze (MWM): A 100 cm pool filled with water rendered opaque with white non-toxic paint was used for all experiments. A 10 cm-diameter escape platform was placed in one quadrant of the pool 1 cm below the surface. During cued trials, a flag was placed on the escape platform to render it visible. Mice underwent 4 trials per day at each of 4 cardinal drop points (north, south, east, west) in random order. If the mouse failed to locate the escape platform within the 60 second trial length, it was manually transferred to the platform for 15 seconds.

Mice were subjected to 3 days of cued trials on post-SAH days 15–17, followed by 4 days of hidden platform trials with the escape platform relocated to a different quadrant on post SAH days 20–23. For hidden platform testing, prominent visual cues were hung at intervals on an opaque, otherwise featureless curtain that surrounded the water maze. At least 10 min elapsed between subsequent trials on a given day. One day after completion of hidden platform testing (post-SAH day 24), the escape platform was removed from the pool and mice were subjected to a single probe trial lasting 60 seconds. Latency to platform (visible or hidden) was calculated as an average of the 4 trials in a given day. Improvement in latency to visible platform was calculated as the decrease in latency between day 1 and day 3 of visible platform testing.

Social interaction (SI): Test mice were singly-housed for 24 hours prior to testing for Crawley’s sociability and preference for social novelty^49^ in a 42 × 70 cm, 3-chamber apparatus as previously described^28^. Briefly, two classes of mice were used: test subjects (SAH or sham) and stimulus mice. Stimulus animals were of the same strain and sex as the subject mice, but were at least twice as old. Test animals were allowed to habituate for 5 min in the testing apparatus, which contained an empty wire containment cup in each of the lateral chambers. The test mouse was then confined to the center chamber, and a stimulus mouse was placed in the left containment cup while a dummy mouse was placed in the right (reversed between test subjects). The barriers were removed and the test mouse was allowed to explore freely for 10 min before being confined to the center chamber again. At this point the dummy mouse was replaced with a novel stimulus mouse, and the test mouse was again allowed to explore for 10 min. Stimulus mice were rotated so that each had at least 30 min break between testing, and none were used for more than 4 test sessions per day. Interaction time was defined as the amount of time the test mouse’s head (identified using the TriWise module in SMART) was within a 1.5 cm ring around the interaction cup. Following SI testing, animals underwent an olfactory test in which a flavored cereal piece (Chocolate Toasted O’s) was buried beneath bedding in a corner of a clean cage. The amount of time required to locate the cereal piece was recorded manually.

Tail suspension (TS): The TS test was performed as described^27, 50^. Briefly, on day 30 post-injury mice were suspended by the tail from a rod 30 cm above the bench surface using adhesive tape. To prevent climbing, the tail was passed through a cardstock paper cone (5.4 cm at the base, 5.5 cm tall, replaced for each animal) before it was attached to the rod. The test was recorded for 6 minutes. Time immobile, defined as motionlessness other than momentum from a prior bout of mobility^27^, was scored by an observer blind to injury status.

### Volumetrics

Thirty days after injury animals were sacrificed by transcardial perfusion of 0.3% heparin followed by 4% paraformaldehyde in buffered saline. Brains were removed and post-fixed in 4% paraformaldehyde in buffered saline for 24 hours followed by equilibration in 30% sucrose for at least 3 days. A freezing sliding microtome (Microm HM 430, ThermoFisher Scientific, Waltham, MA, USA) was used to cut 50 µm coronal sections. Sections were rehydrated and stained with Cresyl violet (FD NeuroTechnologies, Columbia, MD, USA) per manufacturer’s recommendations, and the slides were scanned with a Nanozoomer Whole-Slide Imaging System (Hamamatsu, Hamamatsu City, Shizuoka Pref., Japan) or Axio Scanner (Zeiss, Oberkochen, Germany). Evaluation was conducted on sections taken at 300 μm intervals along the anterior–posterior axis of the brain. Partial brain volume was calculated with ImageJ using 9 contiguous sections per animal covering 2.7 mm from anterior commissure to posterior extent of hippocampus. The images were first thresholded manually to isolate brain parenchyma. The thresholded area was then quantified. Images were prepared for display in Photoshop. Cresyl violet-stained sections taken from animals that survived to study completion were also scrutinized for evidence of secondary infarction. Small lesions potentially consistent with infarction were observed in only 3 instances (10.7% of animals; data not shown).

### Statistical methods

All data was analyzed using Prism 7.0 (GraphPad Software, Inc., La Jolla, CA, USA) and SPSS 22 (IBM, Armonk, New York, USA). In all cases, *P* of <0.05 was considered significant. ***P < 0.001, **P < 0.01, *P < 0.05. *P* values for trends noted in text are given in figure legends. Two-sided tests were used for all comparisons. Single time-point behavioral measurements were compared with a Student’s t-test or Mann-Whitney U test, as appropriate (Shapiro–Wilks W-test used to assess normality). The Holm-Bonferroni correction was applied for multiple comparisons. Task acquisition in the MWM was analyzed with repeated measures ANOVA, with Holm-Bonferroni post-hoc testing. Spearman’s rank correlation coefficient was used to analyze all correlations. Data are presented as mean ± standard error of the mean (SEM).
